# Parasite-mediated disruptive selection in a natural *Daphnia *population

**DOI:** 10.1186/1471-2148-8-80

**Published:** 2008-03-07

**Authors:** Meghan A Duffy, Chad E Brassil, Spencer R Hall, Alan J Tessier, Carla E Cáceres, Jeffrey K Conner

**Affiliations:** 1 W. K. Kellogg Biological Station, Michigan State University, Hickory Corners, MI 49060, USA; 2 Department of Zoology, University of Wisconsin, Madison, WI 53706, USA; 3 School of Biology, Georgia Institute of Technology, Atlanta, GA 30332-0230, USA; 4 School of Biological Sciences, University of Nebraska, Lincoln, NE 68588, USA; 5 School of Integrative Biology, University of Illinois at Urbana-Champaign, Urbana, Illinois 61801, USA; 6 Department of Biology, Indiana University, Bloomington, IN 47405, USA; 7 Division of Environmental Biology, National Science Foundation, Arlington, VA 22230, USA; 8 Department of Plant Biology, Michigan State University, East Lansing, MI, USA

## Abstract

**Background:**

A mismatch has emerged between models and data of host-parasite evolution. Theory readily predicts that parasites can promote host diversity through mechanisms such as disruptive selection. Yet, despite these predictions, empirical evidence for parasite-mediated increases in host diversity remains surprisingly scant.

**Results:**

Here, we document parasite-mediated disruptive selection on a natural *Daphnia *population during a parasite epidemic. The mean susceptibility of clones collected from the population before and after the epidemic did not differ, but clonal variance and broad-sense heritability of post-epidemic clones were significantly greater, indicating disruptive selection and rapid evolution. A maximum likelihood method that we developed for detecting selection on natural populations also suggests disruptive selection during the epidemic: the distribution of susceptibilities in the population shifted from unimodal prior to the epidemic to bimodal after the epidemic. Interestingly, this same bimodal distribution was retained after a generation of sexual reproduction.

**Conclusion:**

These results provide rare empirical support for parasite-driven increases in host genetic diversity, and suggest that this increase can occur rapidly.

## Background

If parasites have large impacts on host fitness, they should play an important role in the evolution of host populations. However, examples of parasite-mediated selection on host populations are limited. Furthermore, most studies document directional selection for resistant host genotypes leading to reduced genetic diversity within populations [1,2]. Yet, many populations contain individuals with a wide range of susceptibilities to infection [3]. How is such diversity maintained in nature? One explanation involves frequency-dependent selection by parasites resulting from host-parasite genotype interactions. In this case, the most common host genotypes are also the most susceptible to parasitism, because the parasite genotypes that specialize on those host genotypes dominate. This leads to parasites driving continual selection against common genotypes, favoring rare genotypes and promoting diversity through time [4]. Alternatively, trade-offs between resistance and other traits could promote diversity [5-9]. With particular trade-offs, theory predicts that parasitism could drive disruptive selection, favoring hosts that are either resistant but have reduced fecundity, or susceptible and highly fecund (where the fitness benefits of increased reproduction outweigh the fitness costs of infection; [5-7,9]). Despite these theoretical predictions, empirical evidence supporting parasitism as a diversity-promoting mechanism in natural populations is surprisingly scant [1,10].

In this study, we look for changes in the susceptibility of a natural population of the freshwater crustacean *Daphnia dentifera *during an epidemic of the yeast parasite *Metschnikowia bicuspidata*. *Metschnikowia *is highly virulent [11,12] and has previously been suggested to affect the evolution of *D. dentifera *populations [2]. We document a large epidemic, during which instantaneous population growth rate (*r*) and infection prevalence were negatively correlated. These pronounced temporal changes in the vital rates of the population suggest the possibility of strong selection. We then use both traditional methods and a novel maximum likelihood approach to document disruptive selection on the host population during the epidemic and to show that the resulting increase in genetic variance was maintained even after a generation of sexual reproduction.

## Results

**Figure 1:**
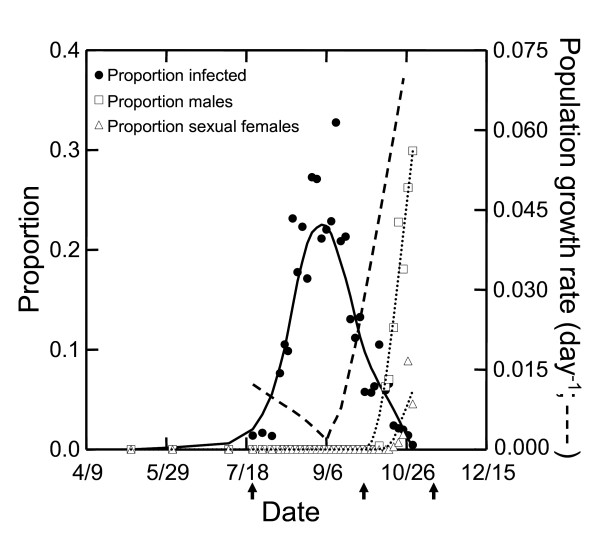
***Daphnia dentifera *population dynamics and infection prevalence**. Left axis: proportion of *D. dentifera *population infected with *Metschnikowia *(circles); proportion of population that are males (squares); proportion of population that are sexual females (open triangles). Right axis: instantaneous population growth rate (*r*; dashed line). Arrows indicate dates on which clones were collected for assays: first arrow: pre-epidemic, second arrow: post-epidemic, third arrow: post-sex sampling. Locally weighted (lowess) smoothing lines are shown.

The *Metschnikowia *epidemic in Bristol Lake in 2004 was large, reaching a maximum infection prevalence of 32% and lasting for more than 100 days (Figure 1). *Metschnikowia *prevalence was negatively correlated with instantaneous population growth rate (*r*; r = -0.65, p < 0.001; Figure 1); *r *decreased 15-fold during the first half of the epidemic and then rebounded rapidly as the epidemic waned, increasing by nearly two orders of magnitude.

**Figure 2:**
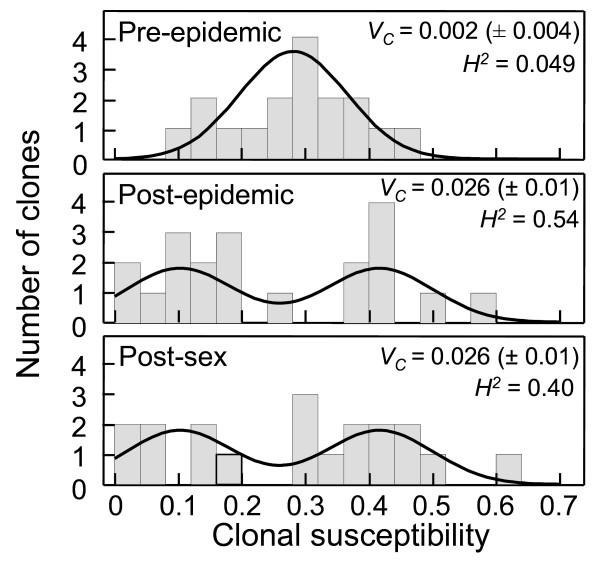
**Distributions of clonal susceptibility in the *D. dentifera *population**. Panels show the distribution of clonal susceptibility before (top panel) and after (middle panel) the epidemic and after sexual reproduction (bottom panel). Histograms showing the experiment data are shown with probability density functions for the best fit model (Table 1) overlain. Probability density functions are scaled to a similar area under each curve as the area of the data in the histograms. Clonal variance (*V*_*C*_; ± 1 standard error) and broad-sense heritability (*H*^2^) estimates are given for each time period.

During this epidemic, the mean susceptibility of clones did not change, but variance increased greatly (Figure 2); together these indicate disruptive selection [13]. The mean pre- and post-epidemic susceptibilities were 0.28 and 0.24, respectively. A planned contrast of these two time periods was not significant (F_1,53 _= 0.50, p = 0.48) despite sufficient power (0.7) to detect a mean change of 0.1. There was a more than threefold increase in variance (randomization test p = 0.004) over the course of the epidemic: the pre- and post-epidemic variances were, respectively, 0.008 and 0.03. This increased variance in resistance was not significantly altered by the episode of sex. There was no significant difference in the mean (F_1,53 _= 0.87, p = 0.35) or variance (p = 0.43) of the post-epidemic and post-sex clones (post-sex mean = 0.29, variance = 0.03), despite sufficient power (0.7) to detect a mean change of 0.12. Clonal variance (*V*_*C*_) and broad-sense heritability (*H*^2^) for susceptibility to infection also increased dramatically between the pre- and post-epidemic time periods (Figure 2), as would be expected with disruptive selection. Clonal variance remained the same after sexual reproduction and heritability decreased slightly due to increased within-clone variance.

Disruptive selection should not only increase a trait's variance but also can change its underlying distribution in a population. More specifically, disruptive selection can lead to a bimodal trait distribution [13]. To better characterize such changes in the distribution of our focal trait (susceptibility to infection), we developed a maximum likelihood method that can detect and differentiate between various forms of selection (in this case, directional and disruptive). The two best fitting models (models 6 and 7; see Table 1 and Methods) both indicated that clonal variance for susceptibility increased over the course of the epidemic. According to model 7, which is more parsimonious and therefore was selected as the best fitting model, the distribution was unimodal prior to the epidemic and bimodal after. In model 6, the distribution is bimodal at both times, but the pre-epidemic modes are quite similar to each other (the 95% confidence intervals overlap completely) while the post-epidemic modes are quite different (the confidence intervals do not overlap at all; Table 1). In both models, the distribution of susceptibilities of the post-epidemic and post-sex clones was the same.

**Table 1:** Models describing the distributions of clonal susceptibility for the pre-epidemic, post-epidemic and post-sex clones.

Type of selection	Model	Pre-epidemic		Post-epidemic		Post-sex		n	AIC	evidence ratio
No selection	1) unimodal: null	*μ*_A_ *σ*_A_		*μ*_A_ *σ*_A_		*μ*_A_ *σ*_A_		2	-45.29	2145
	2) bimodal: null	*μ*_1A_ *σ*_A_	*μ*_2A_ *σ*_A_	*μ*_1A_ *σ*_A_	*μ*_2A_ *σ*_A_	*μ*_1A_ *σ*_A_	*μ*_2A_ *σ*_A_	3	-51.61	90.8
Directional selection	3*) unimodal: mean, var differ	*μ*_A_ *σ*_A_		*μ*_B_ *σ*_B_		*μ*_C_ *σ*_C_		6	-44.74	2817
	4) unimodal: pre-epidemic differs	*μ*_A_ *σ*_A_		*μ*_B_ *σ*_B_		*μ*_B_ *σ*_B_		4	-47.99	557
	5*) bimodal: all modes differ	*μ*_1A_ *σ*_A_	*μ*_2A_ *σ*_A_	*μ*_1B_ *σ*_A_	*μ*_2B_ *σ*_A_	*μ*_1C_ *σ*_A_	*μ*_2C_ *σ*_A_	7	-56.96	6.27
	6) bimodal: pre-epidemic differs	*μ*_1A_ *σ*_A_	*μ*_2A_ *σ*_A_	*μ*_1B_ *σ*_A_	*μ*_2B_ *σ*_A_	*μ*_1B_ *σ*_A_	*μ*_2B_ *σ*_A_	5	-60.55	1.04
**Disruptive selection**	**7) mixed: uni-bi.-bi., same modes**	***μ***_A_ ***σ***_A_		***μ***_1B_ ***σ***_A_	***μ***_2B_ ***σ***_A_	***μ***_1B_ ***σ***_A_	***μ***_2B_ ***σ***_A_	**4**	**-60.63**	**1**
	8*) mixed: uni.-bi.-bi., modes differ	*μ*_A_ *σ*_A_		*μ*_1B_ *σ*_A_	*μ*_2B_ *σ*_A_	*μ*_1C_ *σ*_A_	*μ*_2C_ *σ*_A_	6	-56.86	6.58
	9*) mixed: uni.-bi.-uni.	*μ*_A_ *σ*_A_		*μ*_1B_ *σ*_B_	*μ*_2B_ *σ*_B_	*μ*_C_ *σ*_C_		7	-52.02	74

The mean/mode parameter of the unimodal normal distribution is *μ *and the standard deviation is *σ*. The central tendency parameters for the modes in the bimodal normal distributions are *μ*_1 _and *μ*_2 _and the standard deviation for each mode is *σ*_*i*_. Within each model, parameters with the same subscript are constrained to be equal. Parameters for the best fit model (model 7, highlighted in bold) with 95% confidence intervals based on likelihood profile [51] in parentheses are *μ*_A _= 0.28 (0.24–0.32), *μ*_1B _= 0.42 (0.38–0.46), *μ*_2B _= 0.10 (0.06–0.15), *σ*_A _= 0.08 (0.07–0.11). Parameters for model 6 are *μ*_1A _= 0.21 (0.11–0.40), *μ*_2A _= 0.34 (0.11–0.40), *μ*_1B _= 0.42 (0.38–0.45), *μ*_2B _= 0.10 (0.06–0.14), *σ*_A _= 0.07 (0.06–0.10). Model selection is based on Akaike's Information Criteria (AIC) which incorporates the number of estimated parameters (n). "Uni" = unimodal; "bi" = bimodal. The evidence ratio is the odds ratio of support for the best fit model over the listed model [50]; models with evidence ratios close to 1 are well-supported. Models that allow for a change in the distribution after sexual reproduction have an asterisk (*) after the model number.

## Discussion

Our data suggest that this parasite epidemic caused disruptive selection on the host population, leading to increased genetic variation (i.e., variance among clones) for susceptibility to infection. Examples of disruptive selection of any kind on natural populations are uncommon [14]; to our knowledge, parasite-mediated disruptive selection has never been detected previously. Furthermore, the evolution of this population was rapid, occurring within a 70-day period (about 5–6 asexual generations). Thus, this study supports theoretical predictions [5-7,9] that parasitism can increase genetic variance within a host population, and suggests that this increase can occur rapidly.

Theory indicates that parasitism can promote genetic variation through frequency-dependent selection by the parasite resulting from host-parasite genotype interactions [4]. However, in this case, the most common clones should be the most susceptible and highly susceptible clones should be selected against. Our data do not support either of these patterns: the most common pre-epidemic clones had intermediate susceptibility (Figure 2), and the highly susceptible clones (i.e., those on the right side of Figure 2) increased in frequency. In addition, this mechanism requires host-parasite genotype (GxG) interactions. While these interactions have been found in another *Daphnia*-microparasite system [15,16], we have found no evidence of such interactions in this *Daphnia-Metschnikowia *system [2].

Thus, in this case, the most likely explanation for the changes in clonal frequencies is a trade-off associated with resistance. We propose a trade-off between susceptibility and fecundity, mediated by the relationships between these traits and body size. There is a positive correlation between body size and fecundity in *Daphnia*, including *Daphnia dentifera *[17,18]. In addition, there is a positive relationship between body size of *D. dentifera *and susceptibility to *Metschnikowia *[19]. Therefore, given that natural *Daphnia *populations contain substantial genetic variation for body size [20-22], there should be a negative correlation between resistance and fecundity, which theory predicts can result in disruptive selection [6].

Yet, if there is a trade-off between resistance and fecundity, why weren't all the pre-epidemic clones highly susceptible? This fundamental question motivates much research in evolutionary ecology: how do natural populations retain so much genetic variation in fitness traits? Answers to the general question often focus on correlations with other traits and on how these correlations depend on environmental context [23-25]. In our specific system, both possibilities may be important. First, in *Daphnia*, body size correlates with other traits, particularly with predation by vertebrate and invertebrate predators [26]. Second, environmental conditions in lakes, especially resource levels, change throughout the growing season, and daphniids experience a trade-off in the ability to exploit high- and low-quality food [27]. Thus, correlations between many traits – including susceptibility to parasites, fecundity, and vulnerability to predators – could arise through joint relationships with body size. Moreover, these correlations may change depending on environmental conditions. Given this context-dependency and theoretical predictions that disruptive selection requires particular trade-offs [9,28], we do not expect that disruptive selection will always occur during *Metschnikowia *epidemics. Indeed, a previous study found evidence for directional selection associated with *Metschnikowia *epidemics [2]. The large increase in heritability that occurred during this epidemic would allow the population to respond more quickly to any future directional selection.

Given that there is not a "control" population in which there was not an epidemic, it remains a possibility that the evolution of this population was not in response to *Metschnikowia*. Temporal changes in clonal structure driven by other processes might also explain the observed patterns. While we cannot rule this out with our current data, this seems unlikely. This *Metschnikowia *epidemic was quite large, and had a correspondingly large effect on population dynamics (Figure 1). Evidence from other lake populations suggests that *D. dentifera *populations evolve even in response to much smaller *Metschnikowia *epidemics that do not have detectable effects host population dynamics [2,29]. Therefore, it seems likely that the change in the distribution of susceptibilities of the host population was driven by the large epidemic of this virulent parasite.

Why did the distribution of clonal susceptibilities remain bimodal after sexual reproduction? After all, with random mating, we would expect an increase in the frequency of moderate-susceptibility genotypes. Thus, the retention of the bimodal distribution may indicate assortative mating; however, without knowledge of the genetics underlying resistance to this parasite, this is not certain. If assortative mating did indeed occur in Bristol Lake, the factor by which the resistance genotypes are sorting remains to be determined. Any assortative mating is unlikely to be temporal since the sexually-produced diapausing eggs used here were produced concurrently. Assortative mating could also be based on spatial segregation. However, in our surveys of several lakes, we find little evidence for vertical or horizontal aggregation of infection [30], which would be expected if animals were segregated by susceptibility. Assortative mating based on body size or chemical cues are also possible and warrant further study.

Maintenance of this bimodal distribution after sex could have important implications for maintenance of diversity in *Daphnia *populations. In *Daphnia*, sexually-produced eggs are dormant and are incorporated into an "egg bank" in lake sediments [31]. These egg banks effectively store diversity because diapausing eggs hatch over a series of years, providing a reservoir of genetic variation that can influence the long-term adaptability of the population [31]. Thus, even if parasite-mediated disruptive selection only occurs occasionally in *Daphnia *populations, the increased genetic variance captured in the egg bank may have long-term effects on host populations. A study on a different *Daphnia*-parasite system also suggests that diapausing egg banks should influence the distribution of susceptibilities in the host population over longer time spans [32].

Our study uses a maximum likelihood method to detect disruptive selection and differentiate it from other forms of selection in nature. While traditional statistical tests suggested disruptive selection, the maximum likelihood methods allowed us to more fully characterize the change in the distribution of the trait (susceptibility) in the population. More specifically, our method allowed us to demonstrate that this trait's distribution shifted from unimodal to bimodal, and that the population retained this bimodal trait distribution after a generation of sexual reproduction. Both of these findings are of particular interest in light of predictions of adaptive dynamics theory (which readily predicts disruptive selection leading to bimodal trait distributions within a population; [33-35]), and would not have been detected using earlier methods.

Our approach should be particularly useful for detecting selection on populations for which the common Lande-Arnold [36] selection gradient approach is not feasible. While the Lande-Arnold approach is very powerful and has been used in an extraordinary number of studies, it requires measuring both the phenotype and fitness of individuals in the field [36]. Unfortunately, such data cannot be collected on many organisms, including *Daphnia*, since it is not possible to track them in the environment. Therefore, we propose the maximum likelihood method used in this study as a complementary approach.

## Conclusion

Theory has long predicted an important role of parasites in the evolution and diversification of their host populations. However, surprisingly little empirical evidence supports parasite-mediated enhancement of genetic diversity of hosts. While theory predicts that parasite-driven increases in host diversity can occur as a result of frequency-dependent selection resulting from host-parasite genotype interactions or disruptive selection, only the former had previously been supported empirically (e.g., [37,38]). Here, aided by a generally applicable, maximum-likelihood based technique, we document disruptive selection during an epidemic of a virulent yeast parasite in a natural population of *Daphnia*, leading to increased genetic variance of the host population. Thus, these results provide important empirical support for parasite-mediated disruptive selection leading to increased host diversity.

## Methods

### Study system

Bristol Lake is a moderately productive, thermally stratified, glacial lake located in Barry County in southwest Michigan, USA [39]. *Daphnia dentifera *(formerly *Daphnia galeata mendotae *and *Daphnia rosea*) is a common grazer in planktonic food webs of lakes in temperate North America [40] and is the dominant *Daphnia *species in this lake in summer and autumn. *Daphnia dentifera *hatch out of sexually-produced diapausing eggs in spring and are common throughout summer and autumn [41]. While we have not studied emergence of *D. dentifera *in these specific populations, an earlier study on *Daphnia galeata mendotae *did not find any hatching after June [42]. Sexual reproduction in *D. dentifera *occurs from mid-October through November (Figure 1; [41]).

*Metschnikowia *is a common parasite of *D. dentifera *in lake populations in southwest Michigan, USA, with prevalences as high as 45% [43]. It is highly virulent, greatly reducing the lifespan and fecundity of infected *Daphnia *[2,11,12], and can drive large declines in *D. dentifera *population density [44]. *Metschnikowia*-infected *D. dentifera *are much more susceptible to predation by fish [29], further increasing the death rate of infected hosts relative to uninfected hosts.

### Field sampling

We sampled the *D. dentifera *population in Bristol Lake (Barry County, MI, USA) for infection prevalence and instantaneous population growth rate (*r*) from May–October 2004, following the methods in Duffy et al. [45]. The population was sampled monthly from early May through early July because infections were never observed until late July in previous years. From late July through early August, the population was sampled, on average, every eight days. We increased the sampling frequency to every three days from 8 August through 30 October 2004 to increase our ability to detect population-level effects of the epidemic. Four samples were collected from the lake on each sampling date; each sample integrates whole water column, vertical net tows taken from three spots within the deep basin of the lake. Extensive spatial sampling of parasitism in other lakes has not found evidence of spatial segregation of infected animals [30]. On each date, three of the four samples were used to determine population densities and instantaneous population growth rate (*r*), which was calculated as: (ln *N*_*j *_- ln *N*_*i*_)/(*j*-*i*), where ln *N*_*j *_and ln *N*_*i *_are the natural log of the average densities on sequential sampling days *i *and *j*. Results were smoothed for graphical presentation in SAS (Proc Loess). The fourth sample was used to determine infection prevalence. We examined a random subsample of 400–500 live *D. dentifera *for *Metschnikowia *infections under a stereomicroscope at 25–50× magnification to be able to detect infections at <1% prevalence. A random subsample of 100–300 (including at least 20 infected) *D. dentifera *was examined during periods of high (>10%) infection prevalence because it was easier to accurately assess higher infection prevalence.

We compared the susceptibility of isofemale lines (hereafter: clones) at three time periods to determine whether the susceptibility of the population to infection changed during this epidemic. We compared clones collected 1) at the start of the *Metschnikowia *epidemic; 2) after the epidemic; and 3) after one generation of sexual reproduction. At each of these three time periods, random samples of the *D. dentifera *population were taken from whole-water column, vertical net tows. Adult females carrying eggs were randomly isolated from these samples and used to establish clones on 23 July ("pre-epidemic" clones), 30 September ("post-epidemic" clones) and 11 and 18 November ("post-sex" clones; Figure 1). "Post-epidemic" clones were established prior to the onset of sexual reproduction (including production of males); thus, any differences in susceptibility cannot be explained by differential investment in sexual reproduction [32,46]. To establish post-sex clones, sexually-produced diapausing eggs were collected from females upon egg release. These eggs were later hatched in the lab. A small number (3–4) of clones were lost from each of the three time periods during the pre-experiment period. For the pre-epidemic and post-epidemic clones, we cannot be sure that each clone is unique. Thus, one potential caveat of our field sampling is that multiple individuals of the same clone may have been included in the susceptibility experiments. The effect of this would be to decrease variance in susceptibility. Because the post-sex clones were established using sexually-produced diapausing eggs, these are unique clones. Note that, for all three time periods, we randomly collected adult females, so the clones from each time period should reflect the underlying distribution in the population.

Clones were maintained under standard lab conditions for at least 3 asexual generations prior to the start of the experiment to minimize maternal effects [47]. Twenty *D. dentifera *clones from each of the three time periods were used to assay for susceptibility to infection; due to loss of three pre-epidemic clones and one post-sex clone before completion of the experiment, the final analysis contained 17 pre-epidemic, 20 post-epidemic and 19 post-sex clones. The contrast of pre- and post-epidemic clones tests for selection by the parasite. *Daphnia *are cyclical parthenogens, and because the population only reproduced asexually between the pre- and post-epidemic clone collections (Figure 1), selection on susceptibility to infection between these time periods would change clonal frequencies. The contrast of post-epidemic to post-sex clones tests whether the consequences of any observed selection during the epidemic were maintained after sexual reproduction.

### Infection assays

Infection assays were conducted following the same general protocol as in Duffy and Sivars-Becker [2]. Due to logistical constraints, the experiment was split into two blocks; each block contained approximately half the clones per time period. For each block, five *Daphnia *(6–9 days old) from each clone were distributed to each of six beakers (= 30 animals/clone) filled with 100 ml filtered lake water containing moderate concentrations (1.5 mg dry weight/L) of *Ankistrodesmus falcatus *phytoplankton as a food source. *Daphnia *were then exposed to 100 *Metschnikowia *spores/ml overnight, after which they were moved into beakers containing filtered lake water and high (>2.5 mg/L) food concentrations. The *Metschnikowia *spores were isolated from Bristol Lake and nearby Baker Lake (Barry County, MI, USA); there is no significant difference in the ability of *Metschnikowia *spores isolated from different lakes to infect *D. dentifera *clones [2].

Animals were kept at 20°C throughout the experiment and changed to fresh water halfway through the experiment. We examined animals for infection 11 days post-exposure, at which time infections are clearly visible but have not yet resulted in mortality [2]. Any beakers in which more than two animals died during the experiment were excluded from the analysis, except in the analysis which modeled error terms as binomially distributed (see below). For the main statistical analyses, susceptibility was calculated as the proportion of *Daphnia *in a given beaker that were infected. For graphical presentation (in Figure 2), an overall susceptibility for each of the clones was calculated by taking the proportion of individuals (from all six beakers) that were infected.

Parasite-mediated directional selection should lead to decreases in mean and variance between the pre- and post-epidemic periods. However, disruptive selection should lead to increased post-epidemic variance, and no, or only a small, change in the mean [13]. In general, if mating is random, variance in post-sex clones should increase. However, if starting from a bimodal distribution, random mating should decrease variance in post-sex clones.

### Statistical analysis

Percent infected data from the two blocks were analyzed together because their grand means differed by only 1%. To have maximum power to detect differences between means, we carried out an ANOVA with planned contrasts between the pre- and post-epidemic treatments and the post-epidemic and post-sex treatments (using SYSTAT). We used a randomization test to test for changes in variance. In this test, data from the two time periods (either pre- and post-epidemic or post-epidemic and post-sex) were shuffled, divided into two groups (equal in sample size to the original treatments) and the differences in variances between these randomized groups were calculated. After 1000 such randomizations (in Matlab), we calculated the probability of the observed difference [48]. Clonal variance (*V*_*C*_) and broad-sense heritability (*H*^2^) were estimated using Proc Mixed (SAS 9.1). *V*_*C *_is the among-clone variance; it was estimated using data from each treatment individually, with clone as the only term in the model. Heritability was calculated as clonal variance/total variance.

Disruptive selection can change the underlying distribution of a trait in a population; more specifically, it can lead to a bimodal trait distribution [13]. We developed a maximum likelihood method for characterizing changes in the distribution of a trait, allowing for the detection of different types of selection. To characterize changes in the distribution of clonal susceptibilities in the population, we examined nine models representing three different selection scenarios: null models assuming no change in distribution through time (i.e., no selection; models 1 and 2; Table 1), directional selection (models 3 and 4), and disruptive selection (models 5–9). In models 5 and 6, the distribution is bimodal at all time periods, but the pre-epidemic modes differ from the post-epidemic modes; if the modes become further separated in the post-epidemic period (as compared to the pre-epidemic period) the population has diverged. In models 7–9, the distribution changes from unimodal prior to the epidemic to bimodal after. In addition, models 3, 5, 8 and 9 allow for a change in the distribution after sexual reproduction, as is expected with random mating. Our models of no selection do not incorporate any potential effects of genetic drift, since there are billions of *D. dentifera *in this population.

Candidate models were fit using maximum likelihood methods (see [49]). Best fitting models were selected using Akaike's Information Criteria (AIC; [50]); the model with the lowest evidence ratio has the best support. Confidence intervals were calculated based on the likelihood profile [51]. The analysis was conducted in Mathematica and in R in order to cross-check results and validate code. In order to facilitate use of this method by others, R code is included (see Additional File 1).

The analysis presented in Table 1 utilized normal distributions (unimodal and bimodal) to model the mean infectivity per clone. A normal distribution is presented 1) because of the tradition in evolutionary ecology of analyzing characters which are continuous and normally distributed, and 2) because, in this case, the normal distribution was the distribution most strongly supported by the data according to AIC comparisons. A bimodal distribution is created by weighting 2 unimodal normal distributions. The most general version of the bimodal distribution allows for differential weighting of each mode; however a nested, simpler model assumes equal weighting of each mode – 0.5 times each normal distribution function. The less complex bimodal distribution, with equally weighted modes, was favored by the data using AIC; it is the basis for the bimodal results presented in Table 1. The results are qualitatively similar for the bimodal distribution with unequal mode weighting; that is, the model supported a shift from a unimodal distribution prior to the epidemic to a bimodal distribution after.

The complete analysis was also conducted using a binomial distribution because infectivity is measured on an individual basis with two possible discrete outcomes for each individual, infected or uninfected. An extension of the binomial distribution is the beta-binomial distribution. A beta-binomial distribution assumes a distribution of infection probabilities among individuals instead of a uniform probability for all individuals but still has a discrete outcome per individual of infected or uninfected [52]. The more complex beta-binomial model was not supported by the data, given the sample size, while the nested, less complex binomial distribution was supported. The results of the analysis conducted using the binomial distribution were qualitatively similar to those presented in Table 1; again, the model supported a shift from a unimodal distribution prior to the epidemic to a bimodal distribution after.

## Authors' contributions

MAD conducted the infection assays. CEB designed the maximum likelihood analysis, with assistance from MAD, AJT and JKC; CEB performed the maximum likelihood analysis. SRH carried out the population dynamical study, with assistance from MAD, AJT and CEC. All authors were involved in designing the study, analyzing data, and writing the manuscript. All authors read and approved the final manuscript.

## Supplementary Material

Additional file 1 **Code for maximum likelihood analysis**. Zip file containing R-code for the maximum likelihood analysis. The free statistical program R can be obtained from http://www.r-project.org. The zip file also contains the data files used for the normal and binomial analyses. Click here for file

## References

[B1] Buckling A Rainey PB The role of parasites in sympatric and allopatric host diversification Nature 2002 420 6915 496 499 10.1038/nature01164 12466840

[B2] Duffy MA Sivars-Becker L Rapid evolution and ecological host-parasite dynamics Ecol Lett 2007 10 1 44 53 10.1111/j.1461-0248.2006.00995.x 17204116

[B3] Little TJ The evolutionary significance of parasitism: do parasite-driven genetic dynamics occur ex silico? J Evol Biol 2002 15 1 9 10.1046/j.1420-9101.2002.00366.x

[B4] Lively CM Fox CW, Roff DA, Fairbairn DJ Parasite-host interactions Evolutionary Ecology: Concepts and case studies 2001 New York , Oxford University Press 290 302

[B5] Antonovics J Thrall PH The cost of resistance and the maintenance of genetic polymorphism in host-pathogen systems Proc R Soc Lond B 1994 257 105 110 10.1098/rspb.1994.0101

[B6] Boots M Haraguchi Y The evolution of costly resistance in host-parasite systems Am Nat 1999 153 4 359 370 10.1086/303181 29586625

[B7] Bowers RG Boots M Begon M Life-history trade-offs and the evolution of pathogen resistance: competition between host strains Proc R Soc Lond B 1994 257 1350 247 253 10.1098/rspb.1994.0122 7991634

[B8] Kraaijeveld AR Godfray HCJ Tradeoff between parasitoid resistance and larval competitive ability in Drosophila melanogaster Nature 1997 389 6648 278 280 10.1038/38483 9305840

[B9] Miller MR White A Boots M The evolution of host resistance: Tolerance and control as distinct strategies Journal of Theoretical Biology 2005 236 2 198 207 10.1016/j.jtbi.2005.03.005 16005309

[B10] Buckling A Rainey PB Antagonistic coevolution between a bacterium and a bacteriophage Proc R Soc Lond B 2002 269 1494 931 936 10.1098/rspb.2001.1945 PMC1690980 12028776

[B11] Ebert D Lipsitch M Mangin KL The effect of parasites on host population density and extinction: Experimental epidemiology with Daphnia and six microparasites Am Nat 2000 156 5 459 477 10.1086/303404 29587512

[B12] Hall SR Tessier AJ Duffy MA Huebner M Cáceres CE Warmer does not have to mean sicker: Temperature and predators can jointly drive timing of epidemics Ecology 2006 87 7 1684 1695 10.1890/0012-9658(2006)87[1684:WDNHTM]2.0.CO;2 16922319

[B13] Endler JA Natural Selection in the Wild 1986 Princeton, NJ , Princeton University Press 336

[B14] Kingsolver JG Hoekstra HE Hoekstra JM Berrigan D Vignieri SN Hill CE Hoang A Gibert P Beerli P The strength of phenotypic selection in natural populations Am Nat 2001 157 3 245 261 10.1086/319193 18707288

[B15] Carius HJ Little TJ Ebert D Genetic variation in a host-parasite association: Potential for coevolution and frequency-dependent selection Evolution 2001 55 6 1136 1145 11475049 10.1111/j.0014-3820.2001.tb00633.x

[B16] Ebert D Ecology, epidemiology and evolution of parasitism in Daphnia 2005 Bethesda, MD , National Library of Medicine (US), National Center for Biotechnology Information

[B17] Lynch M Weider LJ Lampert W Measurement of the carbon balance in Daphnia Limnol Oceanogr 1986 31 1 17 33

[B18] Taylor BE Effects of food limitation on growth and reproduction of Daphnia Archiv für Hydrobiologie Beihefte, Ergebnisse der Limnologie 1985 21 285 296

[B19] Hall SR Sivars-Becker L Becker C Duffy MA Tessier AJ Cáceres CE Eating yourself sick: transmission of disease as a function of feeding biology of hosts Ecol Lett 2007 10 3 207 218 10.1111/j.1461-0248.2007.01011.x 17305804

[B20] Spaak P Hoekstra JR Life history variation and the coexistence of a Daphnia hybrid with its parental species Ecology 1995 76 2 553 564 10.2307/1941213

[B21] Spitze K Quantitative genetics of zooplankton life histories Experientia 1995 51 5 454 464 10.1007/BF02143198

[B22] Tessier AJ Young A Leibold M Population dynamics and body-size selection in Daphnia Limnol Oceanogr 1992 37 1 1 13

[B23] Reznick D Nunney L Tessier A Big houses, big cars, superfleas, and the costs of reproduction Trends Ecol Evol 2000 15 10 421 425 10.1016/S0169-5347(00)01941-8 10998520

[B24] Roff DA Fairbairn DJ The evolution of trade-offs: where are we? J Evol Biol 2007 20 2 433 447 10.1111/j.1420-9101.2006.01255.x 17305809

[B25] Sgrò CM Hoffmann AA Genetic correlations, tradeoffs and environmental variation Heredity 2004 93 3 241 248 10.1038/sj.hdy.6800532 15280897

[B26] Zaret TM Predation and freshwater communities 1980 New Haven , Yale University Press 187

[B27] Tessier AJ Woodruff P Trading off the ability to exploit rich versus poor food quality Ecol Lett 2002 5 5 685 692 10.1046/j.1461-0248.2002.00373.x

[B28] Boots M Bowers RG Three mechanisms of host resistance to microparasites - avoidance, recovery and tolerance - show different evolutionary dynamics Journal of Theoretical Biology 1999 201 1 13 23 10.1006/jtbi.1999.1009 10534432

[B29] Duffy MA Hall SR Selective predation and rapid evolution can jointly dampen effects of virulent parasites on *Daphnia* populations Am Nat 2008 171 4 499 510 10.1086/528998 18260781

[B30] Hall SR Duffy MA Tessier AJ Cáceres CE Spatial heterogeneity of daphniid parasitism in lakes Oecologia 2005 143 4 635 644 10.1007/s00442-005-0005-8 15909131

[B31] Hairston NG Jr. Zooplankton egg banks as biotic reservoirs in changing environments Limnol Oceanogr 1996 41 5 1087 1092

[B32] Duncan AB Mitchell SE Little T Parasite-mediated selection and the role of sex and diapause in Daphnia J Evol Biol 2006 19 1183 1189 10.1111/j.1420-9101.2006.01085.x 16780519

[B33] Dieckmann U Doebeli M Metz JAJ Tautz D Adaptive Speciation 2004 Cambridge , Camdridge University Press

[B34] Rueffler C Van Dooren TJM Leimar O Abrams PA Disruptive selection and then what? Trends Ecol Evol 2006 21 5 238 245 10.1016/j.tree.2006.03.003 16697909

[B35] Waxman D Gavrilets S 20 questions on adaptive dynamics J Evol Biol 2005 18 5 1139 1154 10.1111/j.1420-9101.2005.00948.x 16135102

[B36] Lande R Arnold SJ The measurement of selection on correlated characters Evolution 1983 37 6 1210 1226 10.2307/2408842 28556011

[B37] Dybdahl MF Lively CM Host-parasite coevolution: Evidence for rare advantage and time-lagged selection in a natural population Evolution 1998 52 4 1057 1066 10.2307/2411236 28565221

[B38] Lively CM Dybdahl MF Parasite adaptation to locally common host genotypes Nature 2000 405 679 681 10.1038/35015069 10864323

[B39] Cáceres CE Tessier AJ Incidence of diapause varies among populations of Daphnia pulicaria Oecologia 2004 141 425 431 10.1007/s00442-004-1657-5 15375690

[B40] Hebert PDN The Daphnia of North America: An illustrated fauna 1995 University of Guelph , CyberNatural Software

[B41] Cáceres CE Tessier AJ To sink or swim: Variable diapause strategies among Daphnia species Limnol Oceanogr 2004 49 1333 1340

[B42] Cáceres CE Interspecific variation in the abundance, production, and emergence of Daphnia diapausing eggs Ecology 1998 79 5 1699 1710

[B43] Cáceres CE Hall SR Duffy MA Tessier AJ Helmle C MacIntyre S Physical structure of lakes constrains epidemics in Daphnia populations Ecology 2006 87 6 1438 1444 10.1890/0012-9658(2006)87[1438:PSOLCE]2.0.CO;2 16869418

[B44] Duffy MA Selective predation, parasitism, and trophic cascades in a bluegill-Daphnia-parasite system Oecologia 2007 153 2 453 460 10.1007/s00442-007-0742-y 17497181

[B45] Duffy MA Hall SR Tessier AJ Huebner M Selective predators and their parasitized prey: Are epidemics in zooplankton under top-down control? Limnol Oceanogr 2005 50 412 420

[B46] Mitchell SE Read AF Little TJ The effect of a pathogen epidemic on the genetic structure and reproductive strategy of the crustacean Daphnia magna Ecol Lett 2004 7 9 848 858 10.1111/j.1461-0248.2004.00639.x

[B47] Little TJ O'Connor B Colegrave N Watt K Read AF Maternal transfer of strain-specific immunity in an invertebrate Current Biology 2003 13 6 489 492 10.1016/S0960-9822(03)00163-5 12646131

[B48] Anderson MJ Permutation tests for univariate or multivariate analysis of variance and regression Canadian Journal of Fisheries and Aquatic Sciences 2001 58 626 639 10.1139/cjfas-58-3-626

[B49] Hilborn R Mangel M The Ecological Detective: Confronting models with data 1997 Princeton , Princeton University Press 315

[B50] Burnham KP Anderson DR Model Selection and Multimodel Inference: A practical information-theoretic approach 2002 2nd New York , Springer-Verlag 488

[B51] Venzon DJ Moolgavkar SH A method for computing profile-likelihood-based confidence intervals Appl Stat 1998 37 87 94 10.2307/2347496

[B52] Skellam JG A probability distribution derived from the binomial distribution by regarding the probably of success as variables between the sets of trials J R Stat Soc B 1948 10 257 261

